# Serum neutrophil gelatinase associated Lipocalin as a novel biomarker for diagnosing acute pyelonephritis in adults

**DOI:** 10.1038/s41598-025-03646-9

**Published:** 2025-06-04

**Authors:** Ayat Shaban Mousa ElNahal, Ali Ibrahim, Elsayed Abdelhalim, Sally Hassan Essawy, Ahmed behiry, Mo’men Saadoun, Nahla Nosair, Hebatallah Abdelmaksoud Abdelmonsef, Hossam Nabeeh, Tarek Abdelbaky, Diaa-Eldin Taha

**Affiliations:** 1 Medical Microbiology Department, Faculty of Medicine, Kafrelsheikh University, Kafrelsheikh, 33155 Egypt; 2 Urology Department, Faculty of Medicine, Kafrelsheikh University, Kafrelsheikh, 33155 Egypt; 3 Clinical Pathology Department, Faculty of Medicine, Kafrelsheikh University, Kafrelsheikh, 33155 Egypt; 4 Community medicine Department, Faculty of Medicine, Kafrelsheikh University, Kafrelsheikh, 33155 Egypt

**Keywords:** Pyelonephritis, Neutrophil gelatinase-associated Lipocalin, Biomarker, Urinary tract infection, Biomarkers, Urology

## Abstract

To assess the diagnostic value of serum Neutrophil Gelatinase-Associated Lipocalin (NGAL) in adult patients with Acute Pyelonephritis. A total of 132 participants divided into two groups as 66 Hospitalized patients aged ≥ 20 years suspected of having acute pyelonephritis and 66 age and sex matched participants don’t have pyelonephritis. Acute Pyelonephritis was diagnosed in patients with fever, chills or the costovertebral angle tenderness, and bacteriuria higher than 105 CFU/mL without any other focus of infection. No statistically significant differences were observed between the pyelonephritis and non-pyelonephritis groups regarding demographic data or comorbidities. However, the pyelonephritis group exhibited statistically significant differences in Serum NGAL levels, abnormal urine analysis findings, total leukocyte count, and serum C-reactive protein (CRP) levels, with p-values less than 0.001. This study aims to evaluate the potential of Serum NGAL as an early diagnostic biomarker for pyelonephritis, offering a promising tool for timely and accurate diagnosis.

## Introduction

Pyelonephritis is defined as an infection of the upper urinary tract, consisting of the renal parenchyma, calyces, and pelvis^1^. Acute Pyelonephritis (APN) is a common infectious disease at any age that is more likely to occur in young women and older individuals^2,3^. It is estimated that the global annual incidence of APN is 10.5–25.9 million cases, and the lifetime risk of APN in women is 20–35%^3^^,^^4^.

Pyelonephritis can present as an uncomplicated case or complicated. Uncomplicated cases present usually mild symptoms and can be treated as outpatient. On the other hand, complicated pyelonephritis cases usually need hospitalization to avoid life-threatening complications such as sepsis, septic shock, and multiorgan failure. This is more likely to occur in patients with urinary tract obstruction, recent urinary tract instrumentation, or other urinary tract abnormalities, and in patients who are elderly or have diabetes mellitus^5^.

Patients with acute complicated pyelonephritis is characterized by systemic inflammatory signs such as fever tachycardia, tachypnea, nausea, and chills, which are accompanied by laboratory alterations such as leukocytosis or leukopenia; severe lumbar or abdominal pain may also occur^3^. Because of the broad clinical presentation and the non-specificity of symptoms, the most important criterion for the diagnosis of pyelonephritis is the identification of bacteriuria, usually with elevated colony-forming unit counts (greater than 100, 1000 or 100,000 depending on the clinical scenario). Although the specificity of this finding is not high because elderly patients sometimes have asymptomatic bacteriuria. Moreover, obtaining the results of urine culture can take several days; hence, they are not useful for the early diagnosis of pyelonephritis^6,7^. Therefore, new biomarkers which can accurately and rapidly diagnose pyelonephritis are needed.

In recent years, novel biomarkers of renal dysfunction have attracted attention for the diagnosis of UTIs. NGAL is a 25 kDa protein that belongs to the lipocalin. It acts as a transporter for small hydrophobic molecules and is involved in many physiological processes, such as modulating inflammation, innate immune response, and maintaining metabolic homeostasis^8,9^.

NGAL modulates iron transport as part of antibacterial immunity. During inflammatory processes, bacteria synthesize siderophores. NGAL sequestrates siderophores, prevents bacteria from obtaining iron, and thus decreases bacteria growth and multiplication. It is also involved in the activation and transformation of T- cells towards the Th1 type and regulates bacterial clearance from the urinary system. Moreover, NGAL modulates neutrophils functions like maturation, adhesion, phagocytosis, and bacterial killing. It also acts as chemoattractant for neutrophils^10-14^.

Several studies reported a significant increase in the level of NGAL in serum and urine associated with various renal pathological Conditions. Therefore, NGAL has emerged as a potential biomarker for kidney dysfunction and Acute Kidney Injury (AKI)^15-18^.

Although the accuracy of NGAL for acute kidney injury has been reviewed, its utility in the diagnosis of adult APN has not been widely investigated, so the aim of our study to investigate the diagnostic value of Serum NGAL in adult patients with pyelonephritis.

## Materials and methods

### Study ethics and design

This prospective observational study carried out conducted at urology Unit, Faculty of Medicine, Kafr Elsheikh University Hospital between February 2024, and June 2024 after obtaining approval from the Research Ethics Committee, Kafr El-Sheikh University before starting the study (approval number: KFSIRB 200 − 143 and date of final approval:29/1/2024) and approved by clinical trials (NCT06802796) on 25/1/2025.

The details of the study and procedures were explained to all participants, and informed consent was obtained before enrolling in the study.

### Sampling & study participants

The sample size was determined using an online sample size calculator (web)^19^ based on an expected sensitivity of urine NGAL levels in differentiating pyelonephritis from non-pyelonephritis of 66.7% and an expected specificity of 87.0%. With a precision of 10% and a 5% alpha error (95% confidence level), the minimum required sample size was calculated to be 132 participants.

The study was conducted on a total sample of 132 participants divided into two groups as 66 Hospitalized patients aged ≥ 20 years suspected of having pyelonephritis and 66 age and sex matched participants don’t have pyelonephritis. Pyelonephritis was diagnosed in patients with fever, chills or the costovertebral angle tenderness, and bacteriuria higher than 105 CFU/mL without any other focus of infection, and the definition of pyelonephritis was based on urine culture and abdominal ultrasound was used to assess kidney involvement and rule out complications. Cases of prostatitis were excluded from this study based on the presence of typical lower urinary tract symptoms (LUTS) (e.g., dysuria, urgency, pelvic pain). Non pyelonephritis was diagnosed with fever but had an alternative infection focus, such as lower respiratory tract infections (e.g., pneumonia), gastrointestinal infections, or other systemic infections (e.g., sepsis). without costovertebral angle tenderness and bacteriuria. Additionally, abdominal ultrasound in these patients revealed no signs of renal inflammation or structural abnormalities.

### Study procedure

All patients subjected to full history and stress on history of recurrent UTI, family history of renal stones, history of UTI symptoms like dysuria, frequency, urgency, nocturnal enuresis and fever without obvious sources. Also, a full general examination was done and stress on body temperature, throat examination, chest auscultation to detect any focus of fever and blood pressure. abdominal examination was done and stress on palpation on renal angle and on suprapubic area to detect any tenderness and palpation of abdomen to detect any organomegaly. Radiological and laboratory investigation to all participants were done as :,abdominal CT, abdominal US, urine analysis, urine culture, complete blood picture, serum C-reactive protein (CRP) level, serum creatinine and serum neutrophil gelatinase –associated lipocalin (NGAL).

### Urine culture procedures

Midstream urine samples were collected from patients in a sterile container. Samples were centrifuged and sediments were cultured primarily on blood agar and macConkey’s agar by spread plate technique and incubated for 24 h at 37 °C. All microorganisms isolated from positive cultures (≥ 105 CFU/mL) were identified using Gram staining, cultural characteristics, and different biochemical tests.

### Estimations of biomarkers

Venous blood samples were taken under sterile conditions in serum separator tubes from each participant. Samples were centrifuged for 20 min at 2000–3000 RPM, and the serum was stored in 0.5-ml aliquots at −80֗ C pending analysis. Human neutrophil gelatinase-associated lipocalin ELISA Kit from BT LAB^®^ Bioassay technology laboratory was used for the estimation of Serum NGAL. Procedure was done according to the manufacturer’s instructions. Briefly, the microwells of ELISA plate was precoated with Human NGAL antibody. NGAL present in the sample was added and binds to antibodies coated on the wells. And then biotinylated Human NGAL Antibody was added and binds to NGAL in the sample. Then Streptavidin-HRP was added and binds to the Biotinylated NGAL antibody. After incubation, unbound Streptavidin-HRP was washed away during a washing step. Substrate solution was then added, and color developed in proportion to the amount of Human NGAL. The reaction was terminated by the addition of acidic stop solution and absorbance was measured at 450 nm.

### Data management and analysis plan

The data were analyzed using the Statistical Package for the Social Sciences” SPSS 22.0 software (IBM Microsoft). Kolmogorov’s test tested quantitative data normality. Qualitative variables were prescribed using numbers and percent; the Chi-square test was used for analysis or Fisher’s exact test (if more than 20% of the expected cell value is less than 5). Numerical variables were expressed as median (IQR), minimum, and maximum, and the Mann-Whitney U-test and Kruskal–Wallis test were used to compare groups. For multiple comparisons following the Kruskal-Wallis test, the post hoc Dunn test was used. A Receiver Operating Characteristic (ROC) curve was generated to assess the performance of Serum NGAL in predicting pyelonephritis. P-value (< 0.05) was adopted as the level of significance.

## Result

**Fig. 1:**
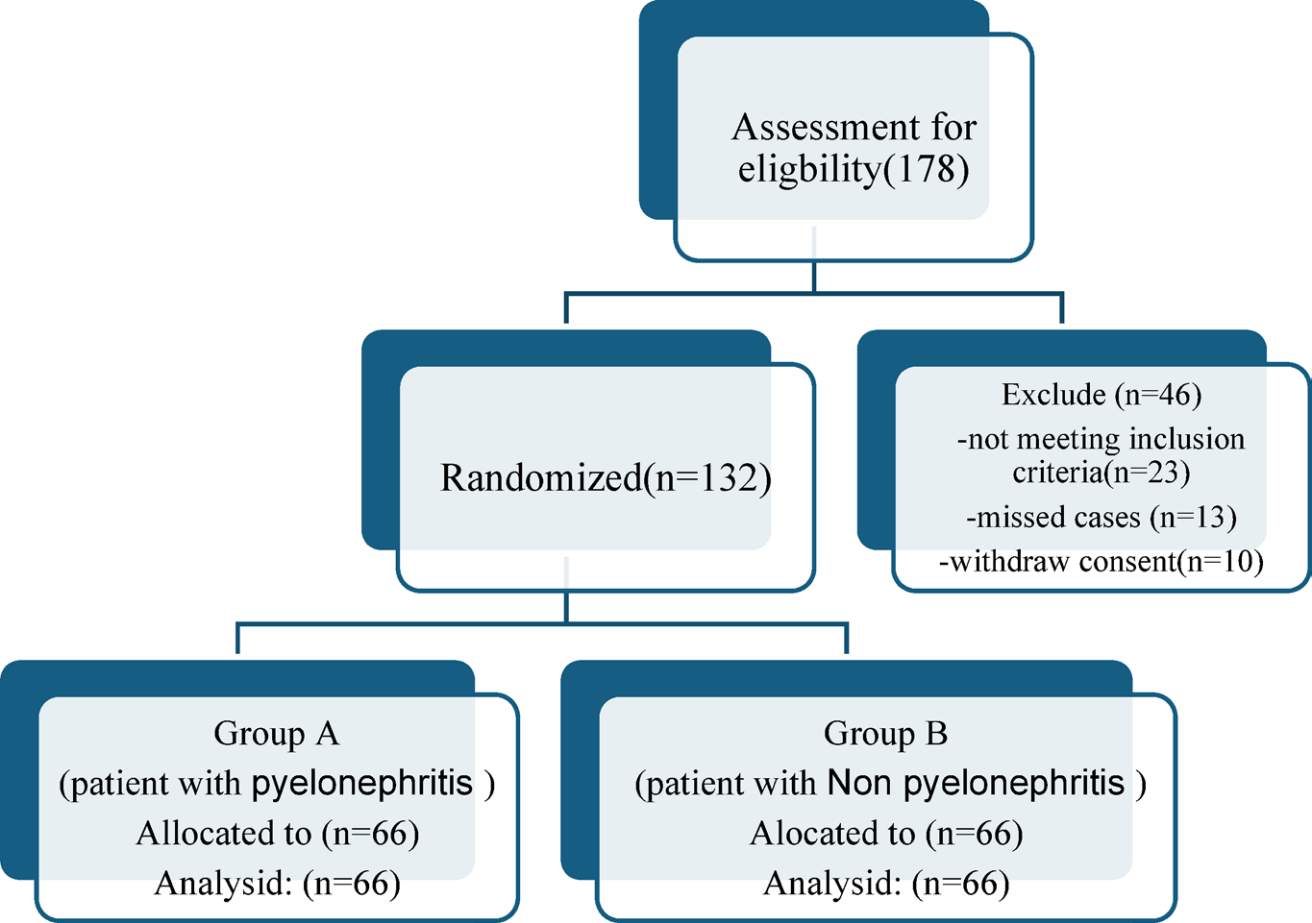
Consort randomization of eligible cases into 2 subgroups.

Out of 178 patients assessed for suitability, only 132 patients enrolled in our study, randomized as 66 patients (50%), 66 patients (50%) in patients with pyelonephritis and patients with Non pyelonephritis group respectively. as shown in the CONSORT chart (Fig. 1).

Regarding to demographic, anthropometric and comorbidity and surgical history among the two groups (Table 1).

- Gender Distribution: No significant difference was found in gender distribution among the groups.
- Age and BMI: No significant difference was found in Age and BMI distribution among the groups.
- comorbidity and surgical history: No significant difference was found in comorbidity and surgical history distribution among the groups.

**Table 1:** Comparison between the studied groups regarding demographic data, anthropometric, comorbidity and surgical history.

Characteristics		Pyelonephritis group (*n* = 66)	Non-pyelonephritis group (*n* = 66)	*P*-value
Age (years)		65(62–70)	67(61–69)	0.573^U^
Gender	Male	56(50.9%)	54(49.1%)	0.640 ^x2^
	Female	10(45.5%)	12(54.5%)	
BMI (kg/m^2^)		24.9(23.0–25.8)	24.3(23.4–25.4)	0.769 ^U^
History of DM		16(59.3%)	11(40.7%)	0.281 ^x2^
History of HTN		23(51.1%)	22(48.9%)	0.854 ^x2^
History of cardiac diseases		5(41.7%)	7(58.3%)	0.545 ^x2^
History of CKD		2(40.0%)	3(60.0%)	1.000 ^FE^
History of hepatic diseases		3(42.9%)	4(57.1%)	1.000 ^FE^
Surgical history		43(48.9%)	45(51.1%)	0.712 ^x2^

*: significant^x2^: chi-squared test ^FE^: Fisher’s exact test ^U^: Mann-Whitney test.

### Laboratory investigations

**Fig. 2:**
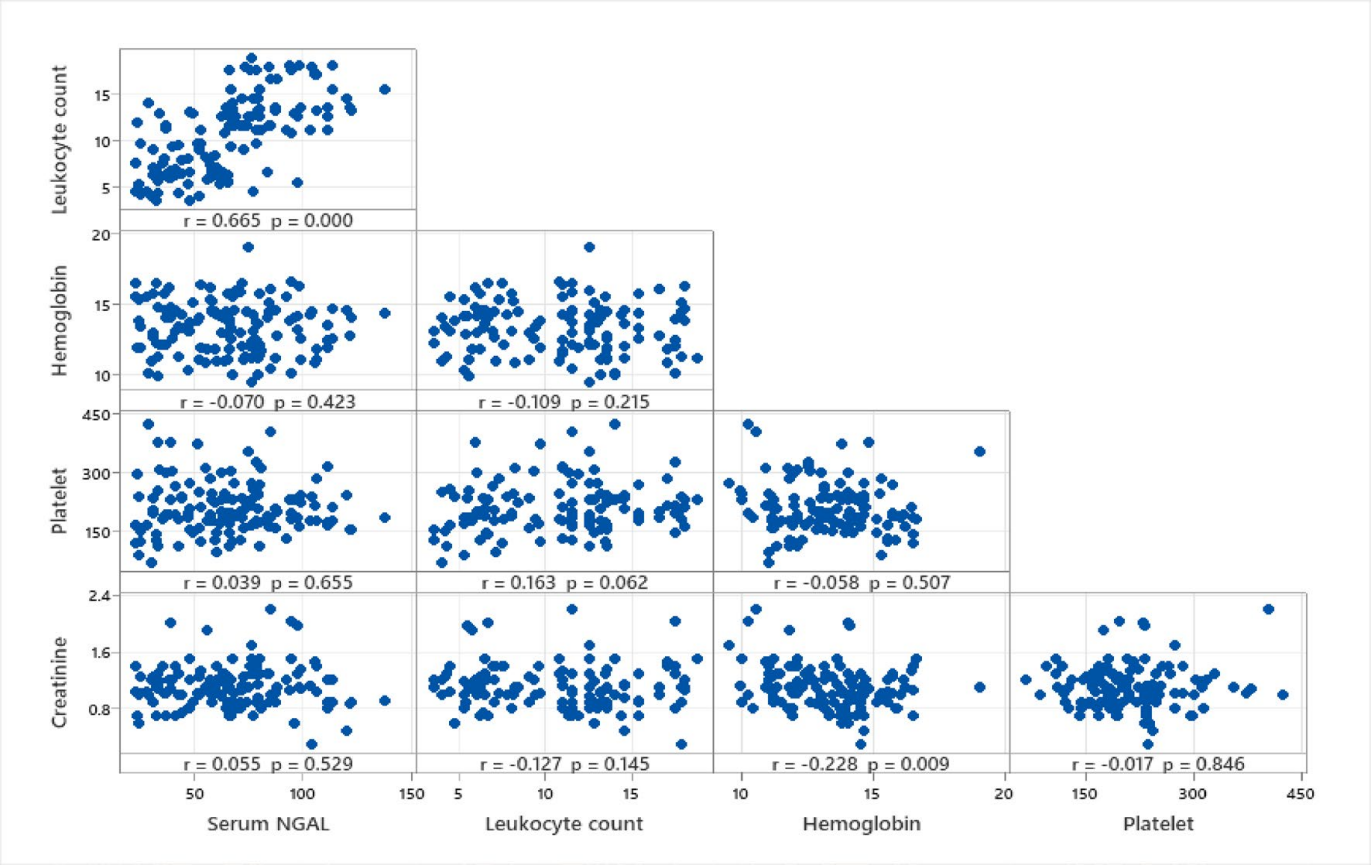
Correlation matrix of serum NGAL, leukocyte count, hemoglobin, platelet count, and creatinine levels.

- Abnormal urine analysis, elevated serum C-reactive protein (CRP), higher leukocyte counts, and elevated Serum NGAL levels show statistically significant more prevalent in the pyelonephritis group with P-value < 0.001 (Table 2).
- But hemoglobin and creatinine level show no statistically significant between two studied groups (Table 2) (Fig. 2).

**Table 2:** Comparison of laboratory investigations between the studied groups.

Investigation	Pyelonephritis group (*n* = 66)	Non-Pyelonephritis group (*n* = 66)	*P*-value
Abnormal Urine analysis	66(100.0%)	0(0%)	< 0.001* ^x2^
Abnormal serum C-reactive protein (mg/L)	80(60–90)	31(13–44)	< 0.001* ^x2^
Leukocyte count (cells/mm³)	13.4(11.5–15.4)	6.5(5.6–8.2)	< 0.001*^U^
Hemoglobin (g/dL)	13.25(11.8–14.6)	13.65(12.1–14.6)	0.662^U^
Platelet (×10^3/µL)	199.5(170–233)	192.0(162–250)	0.818^U^
Creatinine (mg/dL)	1.05(0.9–1.3)	1.1(1–1.25)	0.319^U^
Serum NGAL (ng/mL)	80.0(71.2–98)	45.5(33–59)	< 0.001*^U^

*: significant^x2^: chi-squared test ^U^: Mann-Whitney test.

The correlation matrix provides a comprehensive view of the relationships between Serum NGAL and other laboratory parameters. The strong correlation between NGAL and leukocyte count suggests an association with systemic inflammation, while weaker correlations with hemoglobin, platelet count, and creatinine highlight the specificity of NGAL as a marker distinct from general hematological and renal function indices (Fig. 2).

Regarding distribution of Serum NGAL level across comorbidities

**Fig. 3:**
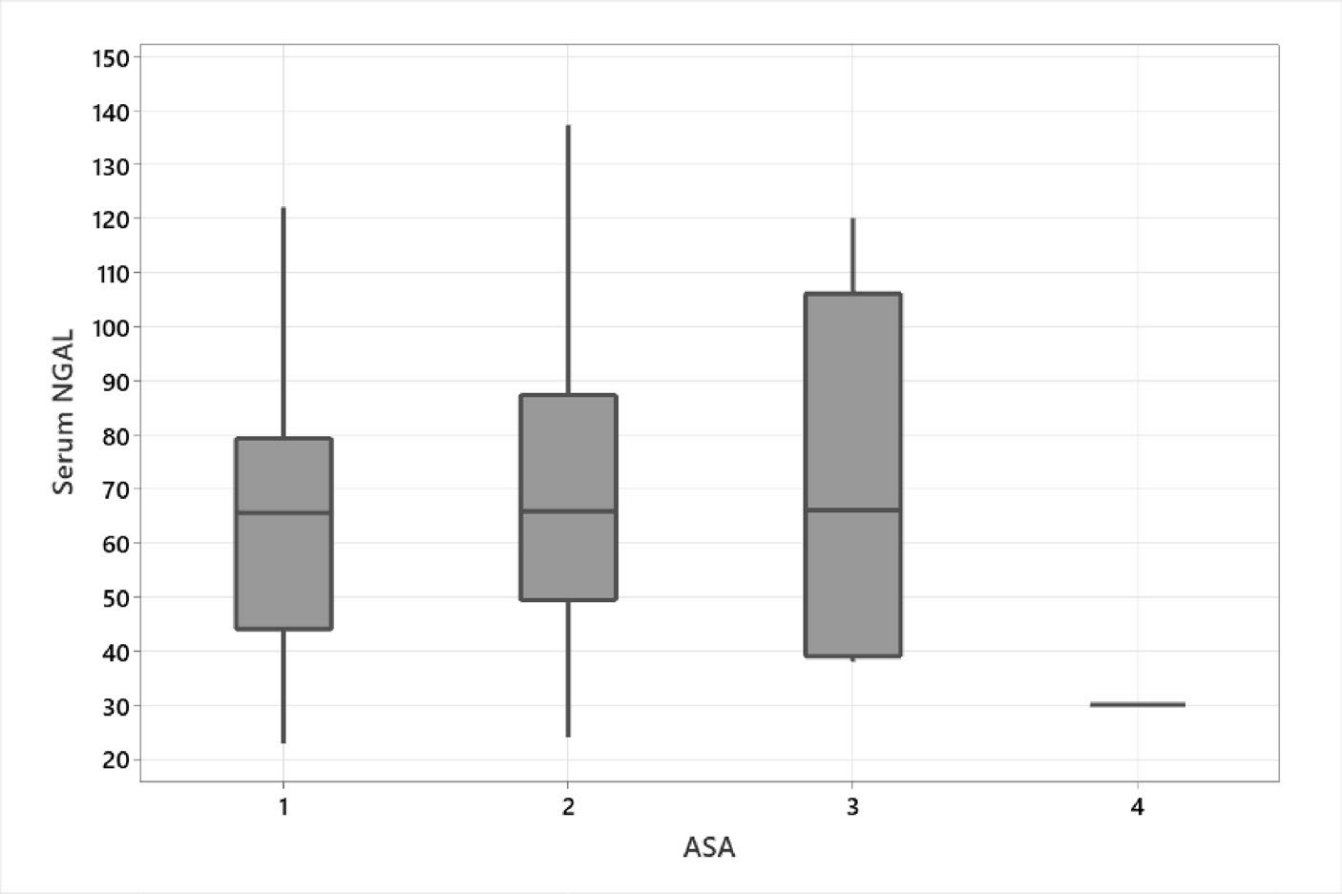
Box plot of serum NGAL levels across ASA classes.

- No statistically significant difference between the studied groups (Table 3) (Fig. 3).

**Table 3:** Distribution of serum NGAL levels across different the American society of anesthesiologists (ASA) classification.

Studied variable		Serum NGAL					P-value
		Minimum	Q1	Median	Q3	Maximum	
ASA	1	22.8	44.0	65.5	79.3	122.0	0.391^K^
	2	24	49.4	65.8	87.3	137.2	
	3	38	39.0	66.0	106.0	120.0	
	4	30	30.0	30.0	30.0	30.0	

*: significant ^K^: Kruskal Wallis test.

Box Plot in Fig. 3 show no significant differences between The distribution of Serum NGAL levels across different ASA classes though the Kruskal-Wallis test indicates. This suggests that NGAL levels may not be strongly influenced by overall patient comorbidity status as classified by ASA. The results support the hypothesis that NGAL is more specific to renal pathology rather than general patient health.

Regarding urine culture,

- There is statistically significant differences in Serum NGAL levels across various urine culture pathogens. Particularly, patients with positive cultures for *E. coli*, *Proteus*, and *Pseudomonas* show markedly higher NGAL levels compared to those with negative cultures (Table 4).

**Table 4:** Comparison of Serum NGAL Levels Across Different Urine Culture Pathogens.

Studied variable		Serum NGAL					P-value
		Minimum	Q1	Median	Q3	Maximum	
Urine culture	E. coli	36.5	67.475	79.5	95.5	137.2	<0.001*^K 1^
	Proteus	71.2	76.75	89.4	104.5	106	
	Pseudomonas	69	75.2	87	101.5	113	
	Others	66	66	72	103	103	
	Negative	22.8	33	45.5	59	97.4	

*: significant ^K^: Kruskal Wallis test ^1^: Pairwise comparisons using a post hoc Dunn test between positive urine cultures (E. coli, Proteus, Pseudomonas) and negative urine cultures revealed statistically significant differences, with adjusted P-values less than 0.001.

These findings further validate NGAL as a sensitive biomarker for detecting bacterial infection in pyelonephritis, with potential implications for pathogen-specific diagnostics.

**Fig. 4:**
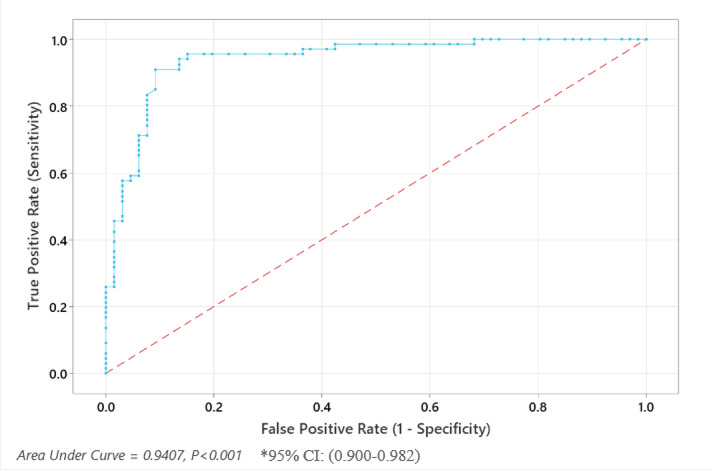
ROC curve showing performance of Serum NGAL in prediction of pyelonephritis in the studied groups.

The ROC curve effectively demonstrates the diagnostic performance of serum NGAL, with a high area under the curve (AUC) indicating strong predictive accuracy for pyelonephritis. According to The ROC curve analysis the optimal cut-off level for predicting pyelonephritis with Serum NGAL level was 62.2 ng/ml. using this cut-off value, the Serum NGAL have sensitivity of 95.5% and specificity of 84.8% for a diagnosis of pyelonephritis.This supports the utility of NGAL as a robust biomarker for distinguishing pyelonephritis from non-pyelonephritis cases, potentially improving diagnostic precision in clinical practice (Fig. 4).

## Discussion

Early detection and proper management of UTIs is important since UTIs involving the kidney may cause permanent renal scarring and be a risk factor for future development of renal insufficiency^20^.

The initial management of acute pyelonephritis (APN) in most patients is primarily based on clinical assessment and laboratory findings. Radiological imaging is typically reserved for more complex cases, including those with atypical symptoms, advanced age, diabetes, immunocompromised status, or underlying congenital abnormalities^21^.

Establishing the diagnosis of acute pyelonephritis (APN) requires imaging with 99 m Tc-dimercaptosuccinic acid (DMSA) scintigraphy to view renal parenchymal involvement^22^. This imaging modality provides visualization of the renal cortex and may reveal peripheral areas of decreased uptake, which are indicative of acute pyelonephritis or scar formation. Currently, its application is primarily limited to pediatric populations for the detection of renal scarring. Scar formation following acute pyelonephritis is uncommon in adults^23^. Although a DMSA scan is considered to be very sensitive in the assessment of renal damage, it is expensive, not readily available in all centers, and exposes the patients to radiation^24^.

Therefore, a more practical and accessible tool to determine the presence of renal parenchymal involvement could be helpful for the timely management of UTIs. NGAL is a 25 kDa protein originally purified from human neutrophils^25^. It is considered a specific marker of neutrophil activity and a strong bacteriostatic agent, as it involves the antibacterial iron-depletion strategy of the innate immune system^26,27^.

NGAL can be tested in both blood and urine. These two forms of NGAL have distinct mechanisms of induction and upregulation, allowing for different applications in clinical settings^28^. pNGAL has been reported to be a useful predictor of systemic inflammation associated with pyelonephritis and damage^29-31^.

As the number of studies demonstrating its usefulness in adults is, so far, limited. And there were many studies which compared urinary NGAL in upper and lower urinary tract infection, So We aimed to assess whether an increased Serum NGAL levels might represent a sensitive marker of APN in adults.

In the current study, we found that there is not statically significant between non pyelonephritis and pyelonephritis group according to demographic data and comorbidities.

Regarding lab results between two groups, we found that Abnormal urine analysis, elevated serum C-reactive protein (CRP), higher leukocyte counts, and elevated Serum NGAL levels are significantly more prevalent in the pyelonephritis group with P-value < 0.001. And there are a strong correlation between NGAL and leukocyte count suggests an association with systemic inflammation.

Which consistent with Sim et al. study, plasma NGAL also significantly correlated with other parameters, including serum CRP and serum levels of leukocyte counts^29^. Also, yun et al. demonstrate same results^32^ Yamamoto et al., was found uNGAL to be higher in adult patients with pyelonephritis compared to those with non-pyelonephritis (302 ng/mL vs. 25 ng/mL, *p*= 0.006)^33^.

Piccoli et al. reported conflicting data for using uNGAL levels to detect APN in 50 adult patients. While no statistical difference in uNGAL levels was found between patients with and without APN, the median uNGAL tended to be higher in patients with magnetic resonance-proven APN^34^.

The ROC curve in our study effectively demonstrates the diagnostic performance of serum NGAL, with a high area under the curve (AUC) indicating strong predictive accuracy for pyelonephritis with the optimal cut-off value 62.2 ng/ml with sensitivity of 95.5% and specificity of 84.8% for a diagnosis of pyelonephritis.

Krzemien et al. found that Serum NGAL cut-off value was 100.8 ng/dl with sensitivity and specificity 82.6%^35^. while Yun found optimal cut-off level of Serum NGAL in pediatric was 267 µg/L showed a sensitivity of 72% and a specificity of 71.4%^32^.

In Kim study, the optimal NGAL cutoff value was 117 ng/ml with sensitivity (86%) and specificity (85%)^31^. Also Sim et al., found the optimal cut-off level for predicting APN with plasma NGAL was 102.5 ng/ml. Using this cut-off value, the plasma NGAL had a sensitivity of 89.1% and a specificity of 71.0% for a diagnosis of APN^29^whereas in Seo study, the ideal cut-off value was 61.0 ng/ml with a sensitivity of 75% and a specificity of 78%^30^.

The variation in cut-off values between the current study and previous research is likely due to whether the comparison was made with healthy individuals or those with non-pyelonephritis infections. While the distinction between healthy individuals and pyelonephritis patients is typically straightforward in clinical practice, differentiating pyelonephritis from infections at other sites can be challenging, particularly when fever is the sole presenting symptom.

Numerous studies have assessed the utility of inflammatory markers in predicting acute pyelonephritis (APN) and have shown that commonly used laboratory parameters, such as white blood cell count (WBC), C-reactive protein (CRP), and erythrocyte sedimentation rate (ESR), exhibit low sensitivity and specificity in accurately predicting acute renal parenchymal involvement^36,37^.

Foster et al. suggested that pNGAL is a good marker of systemic inflammation associated with APN and renal damage, while uNGAL is effective for detecting infection in the genitourinary system. Elevated pNGAL and uNGAL levels suggest the presence of systemic inflammation associated with APN, and low pNGAL and elevated uNGAL levels indicate lower UTI, which has not led to systemic inflammation^28^.

We also found that there is no correlation between plasma NGAL and overall patient comorbidities. The results support the hypothesis that NGAL is more specific to renal pathology rather than general patient health.

Our study illustrates significant differences in Serum NGAL levels across various urine culture pathogens. Particularly, patients with positive cultures for E. coli, Proteus, and Pseudomonas show markedly higher NGAL levels compared to those with negative cultures.

The observed differences in NGAL levels among bacterial subgroups, such as E. coli, Proteus, and Pseudomonas, may reflect distinct pathogen-host interactions. NGAL is part of the innate immune response and plays a key role in iron sequestration by binding bacterial siderophores. E. coli, a known strong siderophore producer, elicits robust NGAL upregulation to limit bacterial iron access. Similarly, Pseudomonas aeruginosa produces high-affinity siderophores (e.g., pyoverdine), which are potent NGAL targets. These mechanisms could explain the elevated NGAL levels observed in infections with these organisms, compared to less virulent or siderophore-poor pathogens^26^.

This result agrees with the findings of Haleyet al.,2024 that all multiplex-PCR positive urine specimens had a biomarker consensus (NGAL, IL-1β, and IL-8) positivity > 70%, ranging from 74% for cases with only emerging uropathogens to 86% for cases with E. coli detected. The biomarker consensus positivity for each subgroup was also significantly higher than that of the multiplex - PCR-negative cases^38^.

Our results agree with Elsadek et al.,2023 study that there was significant increasing in level of Serum NGAL in febrile and non-febrile UTI in comparison with the level of Serum NGAL in healthy control group^39^.

On the other hand, unlike our findings Elsadek et al.,2023 reported that there was statistically non-significant difference between type of bacteria and either serum or urine NGAL in the studied patients^39^.

From a clinical utility perspective, NGAL testing has several advantages. The turnaround time for serum NGAL using ELISA-based methods is typically under 2 h, making it feasible for early clinical decision-making, particularly in emergency or inpatient settings. However, NGAL testing is relatively more expensive than conventional markers such as CRP or WBC, especially in resource-limited settings. Therefore, the cost-effectiveness of incorporating NGAL into standard diagnostic pathways should be evaluated in future studies. Rapid immunoassay platforms, if available, may help reduce costs and facilitate broader implementation^40^.

## Limitaion of study

There are some limitations in our study. We evaluated only Serum NGAL concentration, but not urine NGAL concentration. This was a single-center study with small sample size. We need further studies to evaluate Serum NGAL difference in upper and lower urinary tract infection. Another limitation in our study is that we do not evaluate the cost benefits of using Serum NGAL in diagnosis of APN compared to traditional investigation methods.

Moreover, a key limitation of our study is the lack of external validation for the proposed Serum NGAL cut-off value of 62.2 ng/ml for diagnosing pyelonephritis. While our ROC analysis demonstrated high sensitivity and specificity, the generalizability of this threshold to other populations remains uncertain. Differences in patient demographics, underlying health conditions, and clinical settings may influence NGAL levels and diagnostic performance. Future studies should aim to validate this cut-off in diverse populations, ideally through multi-center cohorts and prospective studies, to ensure its broader applicability and reliability in clinical practice.

## Conclusion

Serum NGAL level is elevated in adult patients with pyelonephritis with high sensitivity and specificity. This study paves the way for Serum NGAL level to be used as a diagnostic biomarker for the diagnosis of pyelonephritis.

## Data Availability

The data is contained within the manuscript, any missing details will be available from the corresponding author on reasonable request.

## Abbreviations

NGAL: Neutrophil gelatinase-associated lipocalin
S/PNGAL: Serum / plasma NGAL
uNGAL: Urinary NGAL
PCR: The polymerase chain reaction
IL: Interleukin
AKI: Acute Kidney Injury
AUC: Area under the curve
CRP: C-reactive protein
CT: Computed tomography
APN: Acute pyelonephritis
DMSA: Di mercapto-succinic acid
